# Defect modes in defective one dimensional parity-time symmetric photonic crystal

**DOI:** 10.1038/s41598-023-48737-7

**Published:** 2023-12-04

**Authors:** Tiecheng Wang, Yong Niu

**Affiliations:** 1 College of Physics and Electronic Engineering, Shanxi University, 030006 Taiyuan, China; 2 Institute of Theoretical Physics, Shanxi University, Taiyuan, 030006 China

**Keywords:** Photonic crystals, Nanophotonics and plasmonics

## Abstract

The introduction of defect layers into one-dimensional parity-time (PT) symmetric photonic crystals gives rise to resonances within the photonic bandgaps. These resonances can be effectively explained by our generalized temporal coupled mode theory. The scattering properties and dispersion relation of defect modes exhibit distinct characteristics compared to conventional one-dimensional Hermitian photonic crystals with defect layers. By tuning the non-Hermiticity or other model parameters, the modulus of the generalized decay rate can be reduced, consequently, the electric field concentrated within the defect layer strengthens. This arises due to the unique band structure of one-dimensional PT-symmetric photonic crystals, which differs significantly from that of traditional one-dimensional Hermitian photonic crystals. Furthermore, the interaction between multiple resonances is investigated through the introduction of multiple defect layers. Our study not only provides insights into resonance phenomena in defective non-Hermitian systems but also contributes to the design of relevant optical resonance devices.

## Introduction

In recent years, the artificial composite structures of Parity-Time (PT) symmetric photonic structures have garnered significant attention and undergone extensive investigation^1–3^, they possess balanced distribution of loss and gain represented by the following refractive index profile $n\left( \varvec{r}\right) =n^*\left( -\varvec{r}\right)$. The exploration of these structures originates from PT-symmetric quantum mechanics^4–7^, which, when extended to optical systems, has unveiled a plenty of intriguing phenomena. Notably, they support unidirectional invisibility^8–12^, wherein the reflectivity is zero for incidence from one side, yet nonzero for incidence from the other side. In addition to that, PT-symmetric optical structures deviate from the normal conservation of flow and instead adhere to a generalized unitarity relation^13^, a characteristic deduced from the property of their transfer matrixes, this relationship further explicates the emergence of anisotropic transmission resonance. Moreover, at specific discrete points, these structures can concurrently function as lasers and coherent perfect absorbers^14–18^, such behavior is illuminated through an examination of their scattering matrixes. Researchers also found other remarkable optical properties, such as unconventional band structures^19^ and Bloch oscillations^20^, and so on.

Resonance^21–24^, as a significant phenomenon pervasive in wave systems, has attracted considerable attention within PT-symmetric photonic structures^25,26^. Prior investigations have primarily focused on scenarios where small loss and gain are segregated into two distinct resonant cavities, and then utilized the conventionally established temporal coupled-mode theory to explore their novel optical properties^27–30^. Recently, we have unveiled that PT-symmetric layered heterostructures can also sustain resonances, moreover, this phenomenon has been effectively elucidated by our proposed generalized temporal coupled mode theory for PT-symmetric resonators^31,32^, which accommodates arbitrary degrees of loss and gain. Within the context of this study, we have identified and examined resonances within one-dimensional PT-symmetric photonic crystals subsequent to the introduction of defect layers. The underlying mechanics of these phenomenon are explicable through our generalized temporal coupled-mode theory.

## one-dimensional PT-symmetric photonic crystal with a single defect layer

**Figure 1:**
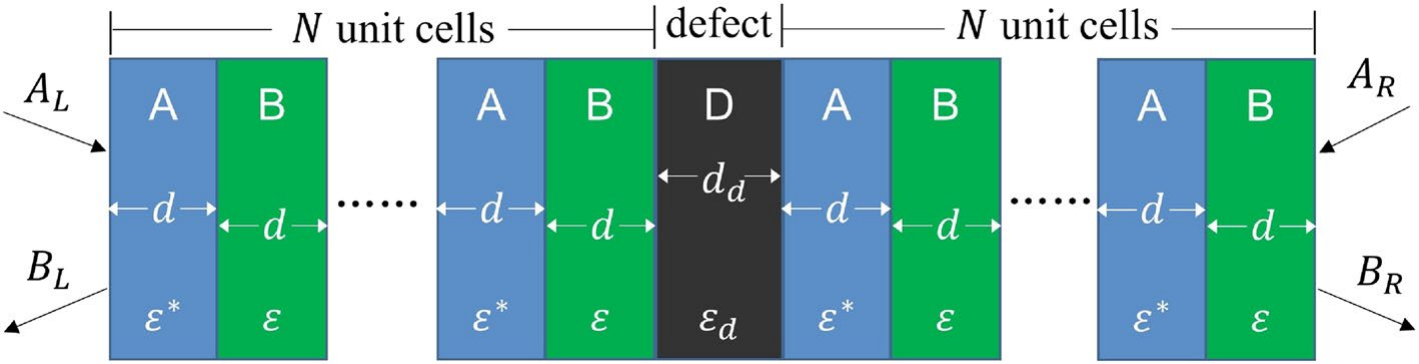
Schematic of one-dimensional PT-symmetric photonic crystal with one defect layer.

Consider the insertion of a defect layer devoid of gain and loss into a one-dimensional PT-symmetric photonic crystal, a representative instance is illustrated in Fig. 1. Here the unit cell of a photonic crystal is composed of two layers with relative permittivities of ${\varepsilon }^*={\varepsilon }_r-i{\varepsilon }_i$ and $\varepsilon ={\varepsilon }_r+i{\varepsilon }_i$, both possessing a thickness of *d*, this results in the unit cell having an overall thickness of $\Lambda =2d$. The defect layer is positioned at the exact center of the photonic crystal to maintain PT-symmetry of the total structure. Its relative permittivity is ${\varepsilon }_d$, with a thickness of $d_d$. The number of unit cells on both sides of the defect layer is denoted as *N*.

**Figure 2:**
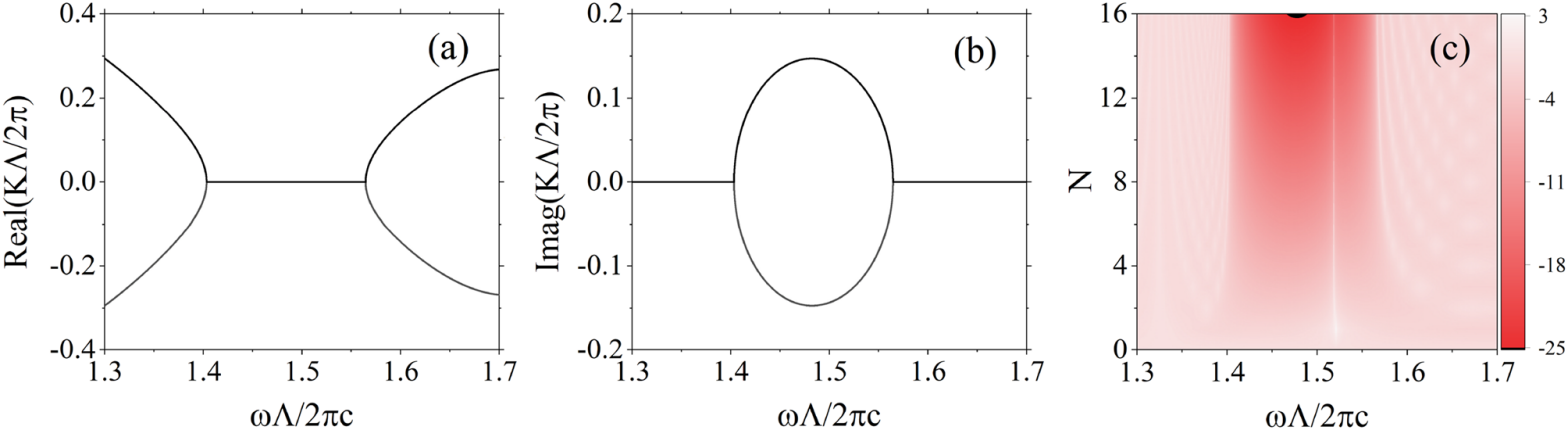
The real part (**a**) and imaginary part (**b**) of the reduced Bloch wave vector as functions of the reduced frequency for the photonic crystal without defect, as well as the variation of the logarithm (base 10) of transmittivity ${\log }_{10}T$ with respect to the reduced frequency and the number of unit cells *N* (**c**) after introducing defect. Here, we employ the transfer matrix method and the chosen parameters are assumed as follows: $\varepsilon =4+1.5i$, ${\varepsilon }_d=6$, $d_d=0.3\Lambda$.

In our investigation of the scattering properties of this structure, we have unveiled the resonance induced by the defect layer, as illustrated in Fig. 2. Figure 2a and b portray the band structure of the photonic crystal in absence of defects, illustrating the variations of the real and imaginary components of the Bloch wave vector with respect to the real frequency. Figure 2c presents the transmittivity spectrum within this frequency range as the number of unit cells *N* are varied. A remarkably sharp resonance peak within the bandgap is clearly discernible in the vicinity of the reduced frequency ${\omega \Lambda }/{2\pi c}=1.519$. The reason why this resonance originates from the defect layer is explained in the section about the dispersion relationships and fied distributions of defect modes.

Next we will employ our generalized temporal coupled-mode theory to analyze the resonance phenomena in the defective structure described above. The foundational and classical coupled-mode theory primarily deals with lossless and gainless Hermitian resonant systems, in our work, the term “generalized” underscores the applicability of this theory to PT-symmetric non-Hermitian resonant systems. This theory, recently proposed by us, aims to explain and facilitate a deeper understanding of resonance in PT-symmetric optical systems. A brief review of this theory is presented below. The temporal evolution of the cavity mode in a resonant system and its input-output relationship can be expressed by the following equations:

1 $$\begin{aligned} \frac{da}{dt}= & {} \left( -i{\omega }_0-\gamma \right) a+{\kappa }^Ts_{+}, \end{aligned}$$

2 $$\begin{aligned} s_{-}= & {} Cs_{+}+da. \end{aligned}$$

Here *i* represents the imaginary unit, $\omega$ stands for the angular frequency of the electromagnetic wave. ${\omega }_0$ is the center frequency of the cavity mode. The total rate is given by $\gamma$, here ${\gamma }>0$ (${\gamma }<0$) denotes that energy dissipates (accumulates) as time increases. Incoming fields from two input channels are marked by the matrix $s_+$, and excite the resonant mode with the coupling matrix $\kappa$. Outgoing fields in two output channels are marked by the matrix $s_-$, and couple with incoming waves not only directly, which is represented by the background scattering matrix *C*, but also indirectly through resonant mode with the coupling matrix *d*.

For a PT-symmetric system, performing a time-reversal transformation is equivalent to a spatial inversion. Consequently, we derive the form of the scattering matrix for a PT-symmetric optical resonant system as follows:

3 $$\begin{aligned} S=C+\frac{2\gamma }{d^T\sigma _xd^*}\frac{dd^T}{-i\left( \omega -{\omega }_0\right) +\gamma }. \end{aligned}$$

Subject to the crucial constraint:

4 $$\begin{aligned} C\sigma _xd^*=-d. \end{aligned}$$

Here $\sigma _x$ represents the first Pauli matrix. Employing this theoretical framework, we elucidate the resonance phenomena in PT-symmetric layered structures. In this structure, the background scattering matrix takes the form of non-transmission:

5 $$\begin{aligned} C=e^{-i\varphi }\left( \begin{array}{cc} b &{} 0 \\ 0 &{} 1/b \end{array} \right) , \end{aligned}$$

where the background transmittivity is zero, indicating no direct coupling between the incident and transmitted waves, the magnitude of the left reflection coefficient of background scattering is *b*, and the magnitude of the right reflection coefficient of background scattering is its reciprocal 1/*b*. This is determined by the generalized unitary relationship satisfied by PT-symmetric layered structures. Substituting this background scattering matrix into the above theory, we can calculate the transmission coefficient, left reflection, and right reflection coefficients as follows:

6a $$\begin{aligned} t= & {} -e^{i\varphi }\frac{\gamma }{\cos \delta }\frac{i}{\omega -{\omega }_0+i\gamma }, \end{aligned}$$

6b $$\begin{aligned} r_L= & {} be^{i\varphi }\frac{\omega -{\omega }_0-\gamma {\tan \delta }}{\omega -{\omega }_0+i\gamma }, \end{aligned}$$

6c $$\begin{aligned} r_R= & {} \frac{1}{b}e^{i\varphi }\frac{\omega -{\omega }_0+\gamma {\tan \delta }}{\omega -{\omega }_0+i\gamma }. \end{aligned}$$

Here, *t* signifies the transmission coefficient, and $r_L$ and $r_R$ represent the reflection coefficients for left and right incidences, respectively. $\delta$ signifies the phase difference between the coupling coefficients of the resonance and the two channels in continuum spectrum. By transforming the background scattering coefficient *b* into its reciprocal and changing the sign of the phase difference $\delta$, the reflection coefficients, $r_L$ and $r_R$ can be interchanged, this can be easily understood by the physical model. It can be readily demonstrated that those scattering coefficients satisfy the generalized unitary relationship applicable to PT-symmetric layered structures: $r_Lr_R=t^2(1-1/{\left| t\right| }^2)$.

On the basis of those coefficients, the transmittivity *T* and reflectivities $R_L$ and $R_R$ for left and right incidences from the view of electromagnetic energy flux can be derived out

7a $$\begin{aligned} T= & {} {\left| t\right| }^2=\frac{{\gamma }^2}{{{\cos }^{2} \delta }}\frac{1}{{\left( \omega -{\omega }_0\right) }^2+{\gamma }^2}, \end{aligned}$$

7b $$\begin{aligned} R_L= & {} {\left| r_L\right| }^2=b^2\frac{{\left( \omega -{\omega }_0-\gamma {\tan \delta }\right) }^2}{{\left( \omega -{\omega }_0\right) }^2+{\gamma }^2}, \end{aligned}$$

7c $$\begin{aligned} R_R= & {} {\left| r_R\right| }^2=\frac{1}{b^2}\frac{{\left( \omega -{\omega }_0+\gamma {\tan \delta }\right) }^2}{{\left( \omega -{\omega }_0\right) }^2+{\gamma }^2}. \end{aligned}$$

Analyzing the scattering rate spectrum where frequency is varied, we observe that the Lorentzian-type transmittivity reaches its maximum value of ${1}/{\cos ^2\delta }$ at the central frequency $\omega ={\omega }_0$. The reflectivity for left incidence reaches its minimum value of zero at $\omega =\omega _0+\gamma {\tan \delta }$, and its maximum value of $b^2(1+{{\tan }^2\delta })$ at $\omega ={\omega }_0-{\gamma }/{\tan \delta }$. The reflectivity for right incidence reaches its minimum value of zero at $\omega =\omega _0-\gamma \tan \delta$, and its maximum value of ${(1+{{\tan }^2\delta })}/{b^2}$ at $\omega =\omega _0+\gamma /\tan \delta$.

The phases of all scattering coefficients can also be analytically investigated. The phase of a complex number, denoted as $c=c_r+ic_i$, can be expressed as $\phi =\theta +\left( \pi \right)$, where $\theta ={\arctan {c_i}/{c_r}}$ and is within the range of $[-\pi /2, \pi /2]$. When $c_r$ is negative, the phase $\pi$ in the parenthesis is considered; conversely, when $c_r$ is positive, the opposite holds true. Hence, employing this approach, the phases ${\phi }_t$ and ${\phi }_{l,r}$ of *t* and $r_{l,r}$, respectively, can be formulated as follows

8a $$\begin{aligned} {\phi }_t= & {} \varphi +\pi +{\theta }_t+\left( \pi \right) , \theta _t={\arctan \frac{\omega -\omega _0}{\gamma }}, \end{aligned}$$

8b $$\begin{aligned} {\phi }_{l,r}= & {} \varphi +{\theta }_{l,r}+\left( \pi \right) , \theta _{l,r}={\arctan \frac{-\gamma }{\omega -\omega _0}}. \end{aligned}$$

For ${\phi }_t$, the phase $\pi$ within the parentheses is considered only when ${\cos \delta }<0$. For ${\phi }_l$, the phase $\pi$ within the parentheses is taken into account only when $\Delta \omega \left( \Delta \omega -\gamma {\tan \delta }\right) <0$. For ${\phi }_r$, the phase $\pi$ within the parentheses is included only when $\Delta \omega \left( \Delta \omega +\gamma {\tan \delta }\right) <0$.

**Figure 3:**
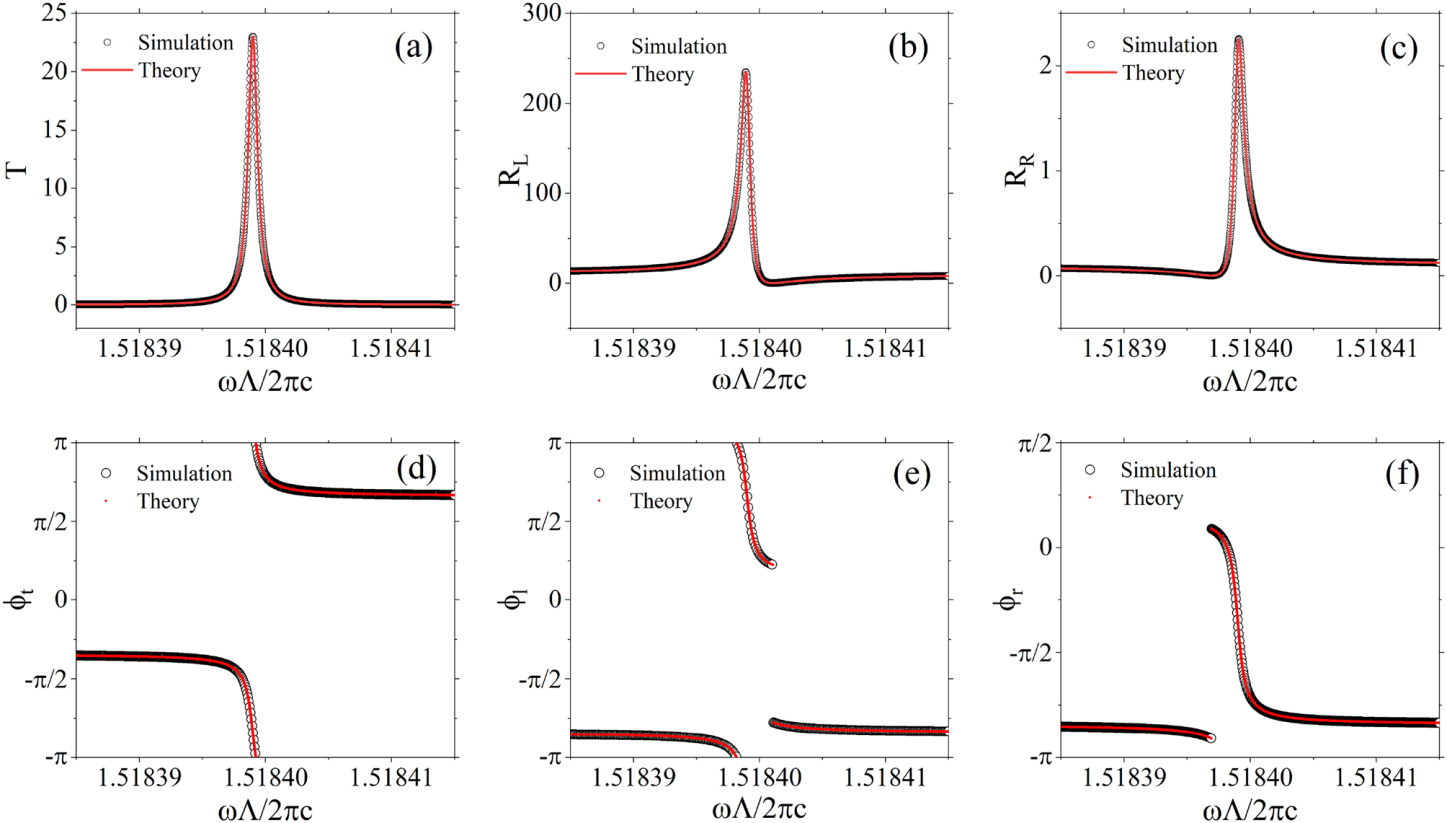
The black circles represent the simulated results obtained through the transfer matrix method, while the red lines and dots correspond to the predictions from our generalized temporal coupled-mode theory. The transmittivity spectrum and the reflectivity spectra for left and right incidences are plotted in (**a**), (**b**), and (**c**), respectively. The phase of the transmission coefficient and the reflection coefficients for left and right incidences are displayed in (**d**), (**e**), and (**f**), respectively. The parameters employed in the fitting theory are as follows: $x=3.1944$, $\varphi =-0.8433\pi$, ${\omega _0\Lambda }/{2\pi c}=1.5184$, ${\gamma \Lambda }/{2\pi c}=-4.4520\times {10}^{-7}$, and $\delta =0.5669\pi$. The remaining parameters of the model remain consistent with those in Fig. 2, and the chosen number of unit cells here is $N=6$.

As evident from Fig. 3, the scattering coefficients obtained by transfer matrix method and our temporal coupled mode theory exhibit a remarkable agreement, thereby providing further evidence of the validity of our theoretical framework. In contrast to the resonances typically observed in conventional photonic crystals, the maxima of transmittivity and reflectivities can surpass unity, even reaching infinity at singular points. This distinction arises from the fact that while conventional photonic crystals are restricted to phase differences of either 0 or $\pi$, these PT-symmetric layered structures accommodate phase differences spanning the interval $[-\pi ,\pi ]$. When the generalized decay rates are negative, both the phase variations of the transmission coefficient and reflection coefficients with respect to frequency are negative. At the central frequency, these phases do not undergo abrupt shifts. This is due to the simultaneous phase jump of ${\theta }_l$ and ${\theta }_r$ by $\pi$, the simultaneous phase jump $\pi$ of the enclosed terms compensates for this effect, resulting in an absence of a net phase jump. At the point of each minimal reflectivity, the phase of each reflection coefficient experiences a jump $\pi$. This is a consequence of the phase jump $\pi$ introduced by the enclosed expression, while ${\theta }_l$ and ${\theta }_r$ experience no phase discontinuities. The aforementioned phenomena are all explicable within the framework of our theory.

## Dispersion relationship and field distribution of defect mode

Resonance arises from the mutual interference between the localized discrete mode of a resonant cavity and the continuous spectrum of its background. The constructive and destructive interferences lead to corresponding maxima and minima in the scattering rates. Subsequently, we investigate the localized states supported by one-dimensional PT-symmetric photonic crystal with defect layer, referred to as defect modes. The formation of these defect modes is attributed to the electromagnetic fields localized in defect layer falling within the bandgap of the photonic crystal, causing the electromagnetic fields to decay in both directions away from the defect layer.

In the following, we quantitatively determine the dispersion relationship of the defect modes. The transfer matrix of the defect layer is denoted as $M_D$, and the transfer matrix of the unit cell of the one dimensional PT-symmetric photonic crystal, represented by *M*, is obtained by electromagnetic boundary conditions at the interfaces and propagations in the dielectrics

9 $$\begin{aligned} M=\frac{1}{8}\left( \begin{array}{cc} 1+k_B/k_0 &{} 1-k_B/k_0 \\ 1-k_B/k_0 &{} 1+k_B/k_0 \end{array} \right) \left( \begin{array}{cc} \left( 1+k_A/k_B\right) e^{ik_Bd} &{} \left( 1-k_A/k_B\right) e^{ik_Bd} \\ \left( 1-k_A/k_B\right) e^{-ik_Bd} &{} \left( 1+k_A/k_B\right) e^{-ik_Bd} \end{array} \right) \left( \begin{array}{cc} \left( 1+k_0/k_A\right) e^{ik_Ad} &{} \left( 1-k_0/k_A\right) e^{ik_Ad} \\ \left( 1-k_0/k_A\right) e^{-ik_Ad} &{} \left( 1+k_0/k_A\right) e^{-ik_Ad} \end{array} \right) , \end{aligned}$$

Here, $k_A$, $k_B$, and $k_0$ are the components of the wave vector in the periodic direction for media A, B, and air, respectively. The widths of media A and B are both *d*. In the calculations, we assume that both sides of each layer of medium have infinitely thin air layers, which do not affect the results and simplify the computation. The relationship between the amplitudes of plane waves propagating forward and backward in each layer of the medium has been established through the use of transfer matrices. The transfer matrix of the unit cell can be diagonalized

10 $$\begin{aligned} M=V\left( \begin{array}{cc} {\lambda }_1 &{} 0 \\ 0 &{} {\lambda }_2 \end{array} \right) V^{-1}, \end{aligned}$$

**Figure 4:**
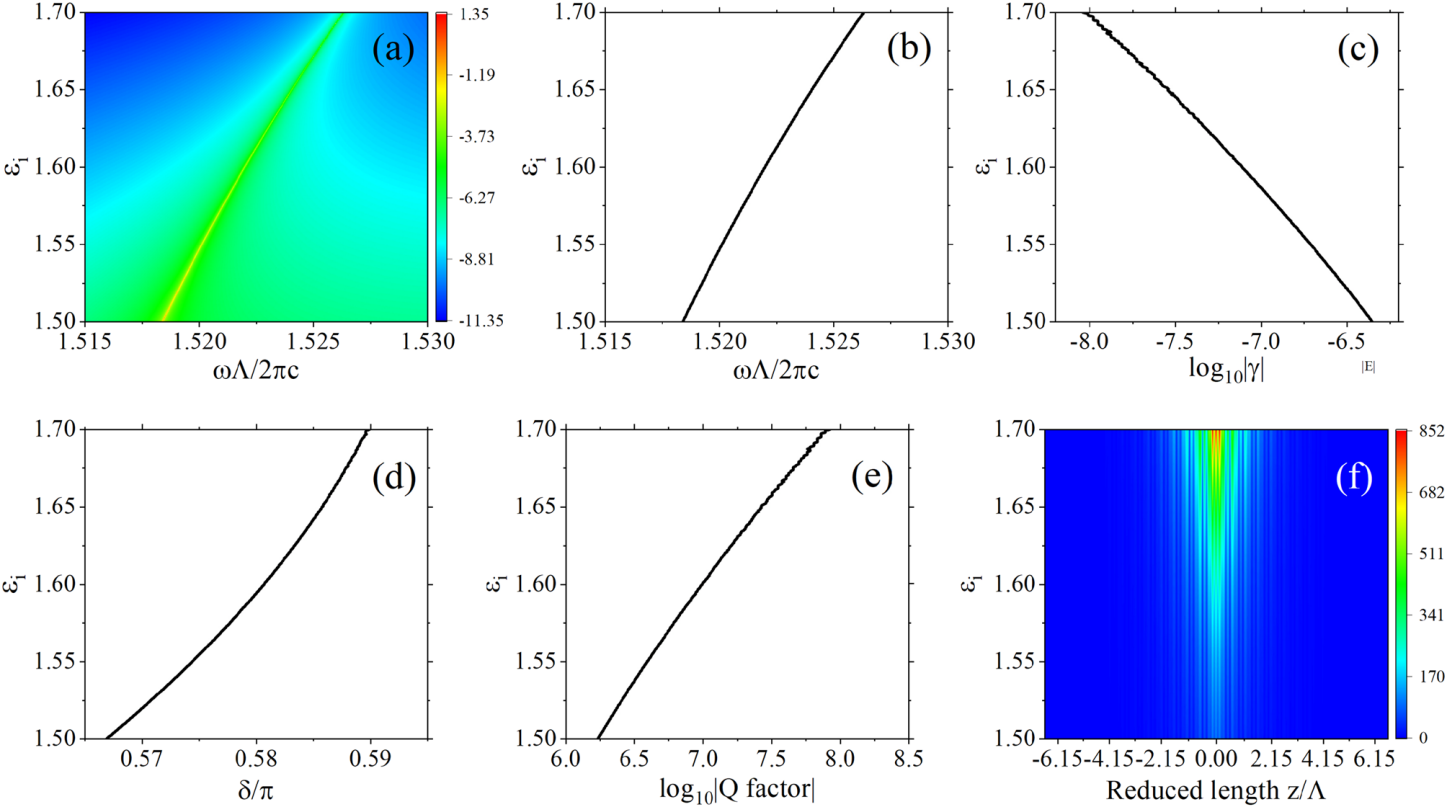
(**a**) The plot of logarithm base 10 of transmittivity spectrum, ${\textrm{log}}_{10}T$, as a function of non-Hermitian degree. (**b**) Dispersion relation of defect modes. (**c**–**e**): Plots of logarithm base 10 of generalized decay rate modulus ${\textrm{log}}_{10}\left| \gamma \right|$, the phase difference $\delta /\pi$, logarithm base 10 of quality factor ${\textrm{log}}_{10}\left| Q\right|$, as functions of non-Hermitian degree. (**f**) Electric field amplitude distribution of defect modes at different non-Hermitian degrees. Each plot uses parameters identical to those in Fig. 3, except for the parameter being studied in that specific plot.

where ${\lambda }_1$ and ${\lambda }_2$ are the eigenvalues of the transfer matrix *M*. In the bandgap, the absolute value of ${\lambda }_1$ is less than 1, while the absolute value of ${\lambda }_2$ is greater than 1. Matrix *V* comprises the two corresponding eigenvectors,the first and second columns corresponds to the eigenvectors of ${\lambda }_1$ and ${\lambda }_2$. Consequently, the overall transfer matrix can be expressed as

11 $$\begin{aligned} \left( \begin{array}{c} A_R \\ B_R \end{array} \right) =V\left( \begin{array}{cc} {\lambda }^N_1 &{} 0 \\ 0 &{} {\lambda }^N_2 \end{array} \right) V^{-1}M_DV\left( \begin{array}{cc} {\lambda }^N_1 &{} 0 \\ 0 &{} {\lambda }^N_2 \end{array} \right) V^{-1}\left( \begin{array}{c} A_L \\ B_L \end{array} \right) . \end{aligned}$$

In order to find the defect mode supporting the resonance, so the electromagnetic transformation is written in this form. Here $M_D$ represents the transfer matrix of the defect layer, which is given by

12 $$\begin{aligned} M_D=\frac{1}{4}\left( \begin{array}{cc} 1+k_D/k_0 &{} 1-k_D/k_0 \\ 1-k_D/k_0 &{} 1+k_D/k_0 \end{array} \right) \left( \begin{array}{cc} \left( 1+k_0/k_D\right) e^{ik_Dd_d} &{} \left( 1-k_0/k_D\right) e^{ik_Dd_d} \\ \left( 1-k_0/k_D\right) e^{-ik_Dd_d} &{} \left( 1+k_0/k_D\right) e^{-ik_Dd_d} \end{array} \right) . \end{aligned}$$

Here $k_D$ and $k_0$ are the components of the wave vector in the periodic direction for the defect layer medium and vacuum, respectively. We set the width of the defect layer to $d_d$. Under the defect mode, the electromagnetic field attenuates in the left and right photonic crystal away from the defect layer. Consequently, the electromagnetic field on the left of the model is in the eigenstate $V_2$ corresponding to ${\lambda }_2$,

13 $$\begin{aligned} \left( \begin{array}{c} A_L \\ B_L \end{array} \right) ={\alpha }_LV_2, \end{aligned}$$

while the electromagnetic field on the right is in the eigenstate $V_1$ corresponding to ${\lambda }_1$,

14 $$\begin{aligned} MV\left( \begin{array}{cc} {\lambda }^N_1 &{} 0 \\ 0 &{} {\lambda }^N_2 \end{array} \right) V^{-1}\left( \begin{array}{c} A_L \\ B_L \end{array} \right) ={\alpha }_RV_1, \end{aligned}$$

note that the eigenstates here are normalized. Combining these two equations yields

15 $$\begin{aligned} MV_2=\alpha V_1, \end{aligned}$$

here $\alpha ={{\alpha }_R}/{\left( {\lambda }^N_2{\alpha }_L\right) }$. Using this relationship, we can determine the dispersion relationship of the defect modes, subsequently obtaining the electric field distributions of the defect modes.

For example, Fig. 4b presents the dispersion relation obtained based on the aforementioned theory. It is evident that it corresponds closely to the resonance spectrum of transmittivity in Fig. 4a. This congruence serves as the evidence that the appearance of resonances within defective one-dimensional PT-symmetric photonic crystals is sustained by discrete defect modes. Figures 4c–e display the variations of generalized decay rate $\gamma$, the phase difference $\delta$ and quality factor *Q* with non-Hermiticity, while Fig. 4f provides the corresponding electric field distribution of the defect mode. These two plots distinctly exhibit that the electric field under the defect mode is concentrated within the defect layer, the generalized decay rate is very low, consequently, a high generalized quality factor. As the degree of non-Hermiticity escalates, the generalized decay rate diminishes, while the concentration of the electric field within the defect layer intensifies. This phenomenon arises due to the reduction of $\left| {\lambda }_1\right|$ alongside the elevation of $\left| {\lambda }_2\right|$ with heightened non-Hermiticity. This leads to a larger amplitude amplification for the eigenstate $V_2$ on the left of the defect layer as it traverses a unit cell of photonic crystal, simultaneously the amplitude reduction of the eigenstate $V_1$ on the right of the defect layer over a unit cell is minimized.

**Figure 5:**
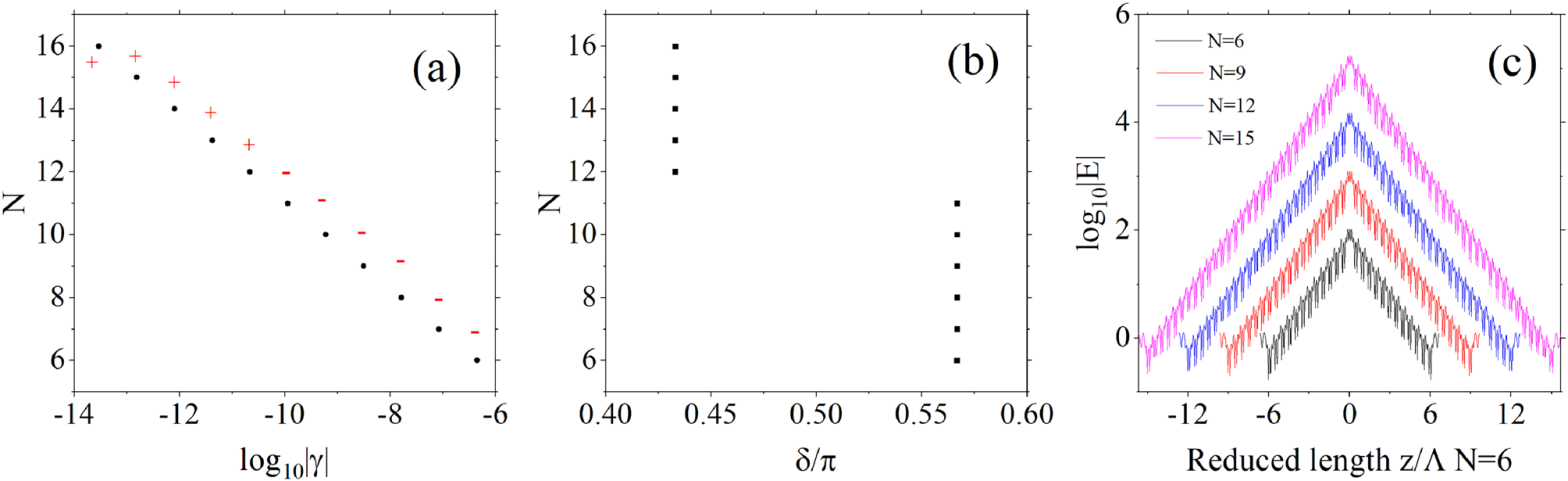
(**a**,**b**): The generalized decay rate ${\textrm{log}}_{10}\left| \gamma \right|$ and the phase difference $\delta /\pi$ for different numbers of unit cells. (**c**) Electric field amplitude distribution of defect modes at different numbers of unit cells. Each plot uses parameters identical to those in Fig. 3, except for the parameter being studied in that specific plot.

Figures 5a displays the generalized decay rate as the number of unit cells changes, the red symbols denotes $\gamma$ is positive or negtive, 5b and c demonstrate the alterations in the phase difference $\delta$ and the defect mode’s electric field distribution as the number of unit cells increases, without modifying the degree of non-Hermiticity. Similar phenomena are discernible, where an increase in the number of unit cells induces a sharp reduction in the generalized decay rate, concomitant with a pronounced enhancement of the electric field concentration within the defect layer. Despite ${\lambda }_1$ and ${\lambda }_2$ remaining constant in this scenario, the augmented unit cells on both sides of the defect layer cause the eigenstate $V_2$ on the left to undergo more amplification cycles, and correspondingly, the eigenstate $V_1$ on the right experiences more amplitude reduction cycles. It worths noting that as the number of unit cells increase the sign of $\gamma$ alters and $\delta$ jumps at the same time, this ensures that the minimum (maximum) of reflectivity locates at the left (right) of center frequency.

The defect modes in one-dimensional PT-symmetric photonic crystals differ significantly from those in conventional one-dimensional Hermitian photonic crystals. By manipulating the non-Hermiticity, the Bloch wave vectors can be distinctly adjusted^19,33^, leading to notable variations in the eigenvalues ${\lambda }_1$ and ${\lambda }_2$ of the transfer matrix *M* of a unit cell. Consequently, the concentration of the electric field of the defect mode within the defect layer can be substantially modified. Exceptional points are unique to non-Hermitian systems^34–36^, near those points resonant singularities of scattering rates arise, where perfect coherent absorption and laser modes are supported. These distinguishing features provide a theoretical foundation for the design of a broader range of potential optical resonators.

## One-dimensional PT-symmetric photonic crystal with multiple defect layers

**Figure 6:**
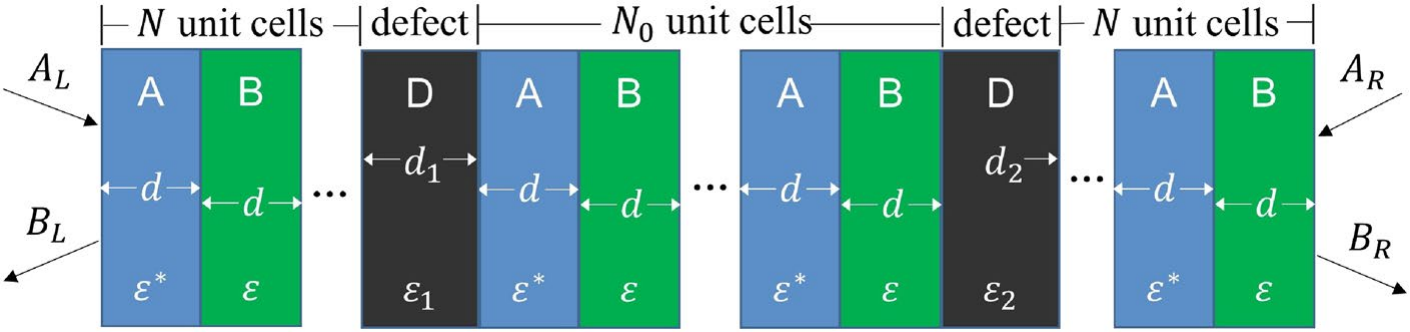
Schematic of a one-dimensional PT-symmetric photonic crystal with two inserted defect layers.

We consider the scenario of inserting multiple defect layers into a one-dimensional PT-symmetric photonic crystal. In this case, each defect layer supports a defect mode. To illustrate this, we examine the case of two defect layers, as depicted in Fig. 6. The interaction between these two defect modes can be also computed using temporal coupled-mode theory, the resonances induced by these two defect modes are governed by the following dynamic equations

16a $$\begin{aligned} \frac{da_1}{dt}= & {} \left( -i{\omega }_1-{\gamma }_1\right) a_1+{\mu }_{12}a_2, \end{aligned}$$

16b $$\begin{aligned} \frac{da_2}{dt}= & {} \left( -i{\omega }_2-{\gamma }_2\right) a_2+{\mu }_{21}a_1. \end{aligned}$$

Here, the absence of incident waves is considered. $a_1$ and $a_2$ represent the amplitudes of the resonances induced by these two defect modes, while ${\mu }_{12}$ and ${\mu }_{21}$ describe their mutual interactions. Assuming that the solutions of the equations take the forms $a_1=A_1e^{\left( -i\omega -\gamma \right) t}$ and $a_2=A_2e^{\left( -i\omega -\gamma \right) t}$, substituting these into the dynamic equations yields

17a $$\begin{aligned}{} & {} \left( -i\omega -\gamma +i{\omega }_1+{\gamma }_1\right) A_1-{\mu }_{12}A_2=0, \end{aligned}$$

17b $$\begin{aligned}{} & {} -{\mu }_{21}A_1+\left( -i\omega -\gamma -i{\omega }_2+{\gamma }_2\right) A_2=0. \end{aligned}$$

The conditions for nontrivial solutions of these equations are derived from the determinant of the coefficient matrix being equal to zero. Consequently, the solutions can be expressed as

18 $$\begin{aligned} \omega -i\gamma =\frac{1}{2}\left( {\omega }_1+{\omega }_2-i{\gamma }_1-i{\gamma }_2\right) \mp \frac{1}{2}\sqrt{{\left( {\omega }_1-{\omega }_2-i{\gamma }_1+i{\gamma }_2\right) }^2-4{\mu }_{12}{\mu }_{21}}, \end{aligned}$$

it can be observed that there are two resonance solutions in general.

**Figure 7:**
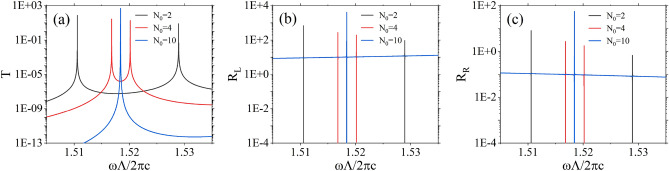
The transmittivity spectrum (**a**), reflectivity spectra for left incidence (**b**) and right incidence (**c**) under different numbers of unit cells $N_0$ between the two defect layers. The black, red, and blue spectral lines correspond to $N_0=2$, 4, and 10, respectively. The two defects are identical, and their parameters are the same as those in Figure 2, as are the other parameters.

We begin our analysis by considering the scenario in which two defect layers are identical. It is evident from the previously discussed method of calculating the dispersion relation of defect modes that, provided the parameters of the defect layers are identical, the dispersion relations of all defect modes are equivalent; conversely, if the parameters differ, the relations are distinct. Therefore, in the absence of mutual interaction between defect layers, ${\omega }_1={\omega }_2={\omega }_d$ and ${\gamma }_1={\gamma }_2={\gamma }_s$. Under these conditions, the resonance solutions can be expressed as

19 $$\begin{aligned} \omega -i\gamma =\left( {\omega }_d-i{\gamma }_d\right) \mp \sqrt{-{\mu }_{12}{\mu }_{21}}, \end{aligned}$$

Figure 7 illustrates the variations in scattering spectra when different numbers of unit cells, denoted as $N_0$, are inserted between two identical defect layers. Notably, for small values of $N_0$, two sharp resonances appear in the photonic bandgap. As $N_0$ increases, the two resonances progressively converge, reducing the separation between them. When $N_0$ becomes significantly large, these two resonances degenerate into a singular resonance. This phenomenon can also be elucidated using the present theoretical framework. When $N_0$ is small, the mutual interaction between the two resonances is substantial, resulting in a significant value for $\sqrt{-{\mu }_{12}{\mu }_{21}}$, leading to the presence of two distinct resonance solutions that exhibit considerable discrepancy. Conversely, for large values of $N_0$, the mutual interaction between these two resonances becomes negligible, rendering $\sqrt{-{\mu }_{12}{\mu }_{21}}$ as zero, thereby resulting in a degeneracy in the two resonance solutions.

**Figure 8:**
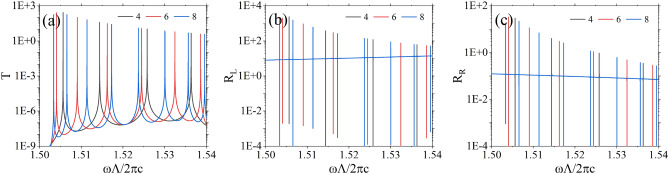
Transmittivity spectra (**a**), reflectivity spectra for left incidence (**b**) and right incidence (**c**) for different numbers of defect layers. The parameters of the defect layers, as well as the parameters of the photonic crystal, are the same as those in Fig. 2.

In Fig. 8 we consider the scenario of inserting more defect layers into a one-dimensional PT-symmetric photonic crystal. Each pair of adjacent defect layers is separated by two unit cells. From this illustration, it is evident that when four, six, or eight defect layers are inserted, there arise four, six, or eight sharp resonances correspondingly within the photonic bandgap. Notably, these resonant peaks exhibit nearly equal frequency spacing, indicating the characteristic traits of comb-like filtering. This phenomenon also finds support in prior theoretical explanations that initially addressed the scenario of two defect layers. Extending this analysis to multiple defect layers employs a similar methodology, consequently, the count of resonances aligns with the number of defect layers present. In Figs. 7 and 8, numerous sharp spectral lines are observed, the transmittivities and reflectivities remain continuous, this phenomenon occurs due to the small decay rate $\gamma$ of the resonant system and the correspondingly high quality factor, the peaks appear very sharp and seem to be discontinuous.

## Conclusion

We have conducted a comprehensive investigation into the resonance phenomena supported by the defect layers inserted into one-dimensional PT-symmetric photonic crystals within the photonic bandgaps. This phenomenon can be elucidated utilizing the generalized temporal coupled mode theory. In the context of resonances supported by PT-symmetric photonic crystals, the phase difference $\delta$ between the coupling coefficients of the resonance and the two channels can take any value within the interval $[-\pi , \pi ]$. The generalized decay rate can even take negative values, which confers upon these resonances exceptional scattering properties not exhibited by conventional Hermitian photonic crystals. Exploiting the characteristics of the photonic bandgap allows us to determine the dispersion relation of the defect mode. In this scenario, the electric field on the left side of the defect layer corresponds to the eigenstate of the eigenvalue ${\lambda }_2$ of the photonic crystal cell transfer matrix with a magnitude greater than one, while the electric field on the right side corresponds to the eigenstate of the eigenvalue ${\lambda }_1$ with a magnitude smaller than one. By adjusting the non-Hermiticity or other model parameters, the magnitude of the generalized decay rate can be diminished, leading to a significant enhancement of the electric field localized within the defect layer. This is attributed to the distinctive band structure of one-dimensional PT-symmetric photonic crystals, where $\left| {\lambda }_1\right|$ is significantly less than one and $\left| {\lambda }_2\right|$ is considerably larger than one. Moreover, the number of defect layers introduced corresponds to the number of resonant phenomena emerging within the photonic bandgap. When the couplings between multiple resonances diminish, degeneracy occurs among the resonances supported by identical defect layers. We anticipate that this work will provide a theoretical foundation for the future design of PT-symmetric resonators with defects.

## Data Availability

Data underlying the results presented in this paper are not publicly available at this time but may be obtained from the calculation by authors, correspondence and requests for materials should be addressed to Tiecheng Wang.
